# Comparison of Anesthesia-Controlled Operating Room Time between Propofol-Based Total Intravenous Anesthesia and Desflurane Anesthesia in Open Colorectal Surgery: A Retrospective Study

**DOI:** 10.1371/journal.pone.0165407

**Published:** 2016-10-25

**Authors:** Wei-Hung Chan, Meei-Shyuan Lee, Chin Lin, Chang-Chieh Wu, Hou-Chuan Lai, Shun-Ming Chan, Chueng-He Lu, Chen-Hwan Cherng, Zhi-Fu Wu

**Affiliations:** 1 Department of Anesthesiology, Tri-Service General Hospital and National Defense Medical Center, Taipei, Taiwan, Republic of China; 2 School of Public Health, National Defense Medical Center, Taipei, Taiwan, Republic of China; 3 Graduate Institute of Life Sciences, National Defense Medical Center, Taipei, Taiwan, Republic of China; 4 Department of Surgery, Division of Colorectal Surgery, Tri-Service General Hospital and National Defense Medical Center, Taipei, Taiwan, Republic of China; Massachusetts General Hospital, UNITED STATES

## Abstract

We conducted a retrospective study to investigate the anesthesia-controlled time and factors that contribute to prolonged extubation in open colorectal surgery. Using our hospital database, demographic data, various time intervals (waiting for anesthesia time, anesthesia time, surgical time, emergence time, exit from operating room after extubation, total operating room time, and post-anesthesia care unit stay time), and incidence of prolonged extubation (≥ 15 mins), were compared between patients who received desflurane/fentanyl-based anesthesia and total intravenous anesthesia via target-controlled infusion with fentanyl/propofol. Logistic regression analyses were performed to assess the association between variables that contributed to prolonged extubation. In conclusion, the anesthesia-controlled time was similar in desflurane anesthesia and propofol-based total intravenous anesthesia for open colorectal surgery in our hospital. Surgical time greater than 210 minutes, as well as age, contributed to prolonged extubation.

## Introduction

Anesthesia-controlled time (ACT) and turnover time (TT) are two of the most important factors that regulate operating room (OR) efficiency [1]. The time required from the end of surgery to extubation is of special interest to surgeons and anesthesiologists because it could be affected by different anesthetic agents or techniques [2–4]. When surgeons scored anesthesiologists’ attributes on a scale from 0 = “no importance” to 4 = “a factor that would make me switch groups/ hospitals”, their average score was 3.9 for “patient quick to awaken” [5]. Accordingly, choosing appropriate anesthetic agents or techniques to avoid prolonged extubation is essential for anesthesiologists in order to improve the efficiency of the OR, even though a small reduction in extubation time may not be sufficient to schedule additional operations, and might reasonably have no economic benefit [6]. Dexter and Epstein (2013) recommended that recording extubation time and monitoring the incidence of prolonged extubation are very important, especially at facilities that have at least eight hours of cases and turnovers [7]. The ACT for total intravenous anesthesia (TIVA) compared with propofol and desflurane (DES) anesthesia was investigated, with controversial results [4,8–16]. After reviewing previous studies comparing extubation times in TIVA with propofol and inhaled anesthesia with DES (Appendix), we found no studies comparing different anesthetic techniques for the improvement of ACT in open colorectal surgery under general anesthesia [8,10–15,17–30]. The majority of previous studies comparing the effects of different anesthesia regimens on OR efficiency have focused on ambulatory or short-duration surgery. Moreover, various propofol delivery techniques, such as target-controlled infusion (TCI) and syringe pump infusion, were used in these studies and may have led to different results. The aim of our present study was to determine whether the use of TIVA with a TCI system is more effective than DES anesthesia in reducing ACT in patients undergoing open colorectal surgery.

## Materials and Methods

**Fig 1:**
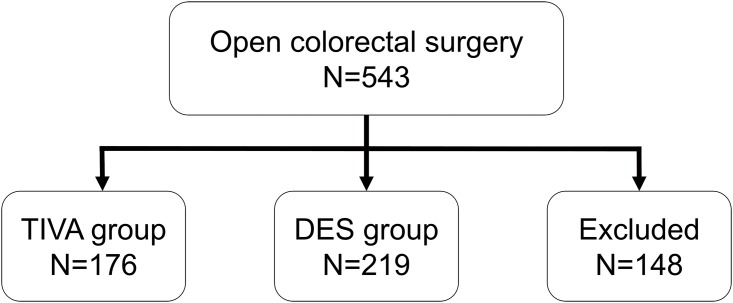
The flow diagram. TIVA = total intravenous anesthesia; DES = desflurane anesthesia.

This retrospective study was approved by the Ethics Committee (TSGHIRB No: 100-05-168) of the Tri-Service General Hospital, Taipei, Taiwan (Chairperson, Professor Pauling Chu) on the 29^th^ of August, 2011. The information was retrieved from medical records and the electronic database of the Tri-Service General Hospital (TSGH; Taipei, Taiwan, Republic of China). We enrolled 395 patients (American Society of Anesthesiologists [ASA] class I–III) who received elective open colorectal surgery under TIVA with TCI (TIVA group, n = 176) or desflurane anesthesia (DES group, n = 219) from January 2010 to December 2011. One-hundred and forty-eight patients were excluded from the analysis. Exclusion criteria were: combined TIVA with inhalation anesthesia or epidural anesthesia (n = 95), other inhalation anesthesia besides desflurane (n = 26), patient not sent to the post-anesthetic care unit (PACU) (n = 15), or incomplete data (n = 12) (Fig 1). We also recorded the patients’ demographic data and American Society of Anesthesiologists (ASA) physical status.

### Anesthetic techniques

There was no premedication before induction of anesthesia. Regular monitoring, such as noninvasive blood pressure, electrocardiography (lead II), pulse oximetry, and end-tidal carbon dioxide pressure (EtCO_2_), was performed for each patient. Anesthesia was induced with fentanyl, propofol, and rocuronium in all patients. The patients were then intubated and maintained with the anesthetics propofol or DES, and the analgesic fentanyl. All time intervals (duration of waiting for anesthesia, duration of anesthesia, surgical time, emergence time, exit from OR after extubation, total OR time, and PACU stay time) were documented as electrical medical records by an perioperative nurse and were confirmed with the operator and the presiding anesthesiologist.

In the TIVA group, anesthesia was induced with intravenous (i.v.) fentanyl (2 μg/kg) and 2% lidocaine (1.5 mg/kg). Continuous infusion of propofol (Fresfol 1%) was delivered subsequently using Schneider’s kinetic model of TCI (Fresenius Orchestra Primea; Fresenius Kabi AG, Bad Homburg, Germany) with an effect-site concentration (Ce) of 4.0 μg/mL. Rocuronium (0.6 mg/ kg) was administered when patients lost consciousness, followed by tracheal intubation. Anesthesia was maintained by using TCI with propofol Ce 3–4 μg/mL and an oxygen flow of 0.3 L/min with FiO_2_ 100%. Repetitive bolus injections of cisatracurium and fentanyl were administered as required throughout the procedure [31].

In the DES group, patients were induced with i.v. fentanyl (2 μg/kg), 2% lidocaine (1.5 mg/kg), and propofol (1.5–2 mg/kg). When patients lost consciousness, 0.6 mg/kg of rocuronium was administered, followed by endotracheal intubation. Anesthesia was maintained by providing 8%–12% desflurane (inhaled concentration) in a 100% oxygen flow of 300 mL/min under a closed system. Repetitive bolus injections of cisatracurium and fentanyl were administered as required throughout the procedure.

Maintenance of Ce for TCI with propofol and DES concentration was adjusted at the range of 0.2 μg/mL and 0.5%, respectively, according to the hemodynamics. If two increments or decrements were unsuccessful, the range of Ce for TCI propofol and desflurane was increased to 0.5 μg/mL or 2%, respectively. The EtCO_2_ pressure was maintained at 35–45 mmHg by adjusting the ventilation rate and maximum airway pressure. Once neuromuscular function was restored, cisatracurium (2 mg) was administered intravenously as required.

The Ce of propofol or DES concentration was tapered to 2.0 μg/mL or 5%, respectively, at the beginning of skin closure. At the last five stitches of surgery, propofol or DES was discontinued, but the oxygen flow was kept at 300 mL/min. At the end of the skin closure, the lungs were ventilated with 100% oxygen at a fresh gas flow of 6 L/min. Reversal of neuromuscular function was achieved by administrating neostigmine (0.03–0.04 mg/kg) with glycopyrrolate (0.006–0.008 mg/kg) once spontaneous breathing returned, to prevent residual paralysis. When the patient regained consciousness (assessed by an anesthesiologist with voice and gentle prodding) with spontaneous and smooth respiration, the endotracheal tube was removed. Then the patient was sent to the PACU for further care. An extubation time (from the end of skin closure until extubation) of 15 minutes or longer was considered prolonged extubation [3].

### Statistical Analysis

Data are presented as the mean and standard deviation (SD), number of patients, or percentage. Demographic and perioperative variables were compared using Student’s t-tests. Categorical variables were compared using chi-square tests. Univariate and multivariate logistic regression analyses were performed to assess the association between variables that contributed to prolonged extubation. Linear trend was tested by treating the quintile grouping as a continuous variable, which was 1 to 5 for quintile 1 to quintile 5, respectively, in the logistic regression model. We classified age and BMI into five equal groups (quintiles) to explore the relationships between the variables and prolonged extubation. To ensure there were enough cases of prolonged extubation in each group, surgical time was classified into three groups: ≤ 210 minutes, 211–240 minutes, > 240 minutes. The level of statistical significance was determined as P < 0.05.

## Results

Our study included 395 patients, of which 219 received DES and 176 received TIVA anesthesia. In order to guarantee unbiased results, the confounding effects of patient characteristics (ASA, gender, age, height, and weight), and various time intervals were investigated. In addition, the amounts of opioids, non-depolarizing muscle relaxants (NDMRs) and reversal agents administered during the surgical period were compared. There were no significant differences in patient demographics (Table 1). The amount of opioids and NDMRs were significantly higher in the TIVA group than in the DES group, while there were no significant differences in reversal agents between the groups (Table 2). There were no significant differences between groups in time waiting for anesthesia, surgical time, anesthesia time, extubation time, exit from OR after extubation, total OR time, PACU time, or the incidence of prolonged extubation (Table 3). The incidence of prolonged extubation showed no significant differences between groups (DES: 14.2%; TIVA: 9.7%; P = 0.17).

**Table 1:** Comparison of patient characteristics.

	Group DES (n = 219)	Group TIVA (n = 176)	P value
ASA II/III	148/71	125/51	0.13
Gender (Male/Female)	123/96	98/78	1.00
Age (y/o)	63.5 ± 11.5	65.0 ± 10.0	0.16
Height (cm)	163.1 ± 8.0	162.8 ± 8.3	0.70
Weight (kg)	63.7 ± 12.5	63.6 ± 12.3	0.94
BMI (kg/m^2^)	23.8 ± 3.7	23.9 ± 3.8	0.84

DES, desflurane anesthesia; TIVA, total intravenous anesthesia; ASA, American Society of Anesthesiologists; BMI, body mass index.

Data are shown as mean ± standard deviation or number.

**Table 2:** Comparison of the amount of opioid, non-depolarizing muscle relaxants, and reversal agents used during surgical periods between the DES and TIVA groups.

	Group DES (n = 219)	Group TIVA (n = 176)	P value
Fentanyl (μg/kg)	2.9 ± 0.7	4.5 ± 1.0	<0.001
Cisatracurium (mg/kg)	0.2 ± 0.1	0.3 ± 0.1	<0.001
Neostigmine (μg/kg)	32.2 ± 4.4	33.0 ± 4.4	0.08
Glycopyrrolate (μg/kg)	6.4 ± 0.9	06.5 ± 0.9	0.08

DES, desflurane anesthesia; TIVA, total intravenous anesthesia.

Data are shown as mean ± SD or number.

**Table 3:** Comparison of operating room time between the DES and TIVA groups.

	Group DES (n = 219)	Group TIVA (n = 176)	P value
Waiting for anesthesia time (min)	7.8 ± 3.5	7.7 ± 3.7	0.81
Surgical time (min)	178.7 ± 45.7	180.1 ± 42.7	0.77
Anesthesia time (min)	214.6 ± 46.7	214.1 ± 45.1	0.90
Extubation time (min)	9.8 ± 4.4	9.5 ± 3.8	0.39
Exit from operating room after extubation (min)	9.4 ± 2.7	9.2 ± 2.7	0.63
Total operating room time (min)	231.8 ± 47.0	230.8 ± 46.1	0.84
PACU time (min)	49.8 ± 12.3	49.9 ± 11.7	0.94

DES, desflurane anesthesia; TIVA, total intravenous anesthesia; PACU, post-anesthesia care unit.

Data are shown as mean ± SD or number.

The results of univariate and multivariate logistic regression analyses comparing prolonged extubation time with several variants in all patients are shown in Table 4. After adjustment for potential covariates (gender and body mass index (BMI)), surgical time (P for trend < 0.001) and age (P for trend = 0.033) were found to independently predict prolonged extubation in a dose-response manner. Surgical times > 210 minutes and age contributed to prolonged extubation, while gender and BMI of the patients had no significant influence.

**Table 4:** Univariate and multivariate logistic regression analyses of variables associated with prolonged extubation in all patients (n = 395).

Variables	n_1_/n_2_	Crude Model			Multivariable Model		
		Odds ratio	95% CI	p value	Odds ratio	95% CI	*p* value
Surgical time (min)							
≤ 210 (ref.)	15/300	1.00			1.00	-	-
211–240	14/39	7.18	3.22–16.0	<0.001	8.10	3.51–18.7	<0.001
> 240	19/8	47.5	17.9–126	<0.001	64.7	21.8–192	<0.001
*p* for trend				<0.001			<0.001
Women (ref: Men)		0.66	0.35–1.24		0.68	0.31–1.46	0.32
Age, y/o							
Q1: 29–55 (ref.)	8/80	1.00	-	-	1.00	-	-
Q2: 56–60	7/65	1.08	0.37–3.13	0.89	1.44	0.40–5.21	0.58
Q3: 61–68	14/69	2.03	0.80–5.12	0.13	2.05	0.62–6.73	0.24
Q4: 69–75	11/68	1.62	0.62–4.25	0.33	2.37	0.67–8.24	0.17
Q5: 76–86	8/65	1.23	0.44–3.46	0.69	3.19	0.90–11.3	0.07
*p* for trend				0.46			0.03
BMI (kg/m^2^)							
Q1: 9.91–20.86 (ref.)	6/72	1.00			1.00	-	-
Q2: 20.87–22.83	12/67	2.15	0.76–6.05	0.15	2.35	0.65–8.43	0.19
Q3: 22.84–24.49	8/71	1.35	0.45–4.10	0.59	1.47	0.38–5.60	0.58
Q4: 24.50–26.67	13/68	2.29	0.83–6.38	0.11	2.50	0.72–8.75	0.15
Q5: 26.68–39.14	9/69	1.57	0.53–4.63	0.42	3.32	0.93–11.9	0.07
*p* for trend				0.46			0.09

CI: confidence interval; P value < 0.05 were considered significant. Q: quintile.

n_1_: number of patients with prolonged extubation in each group; n_2_: number of patients without prolonged extubation in each group.

## Discussion

In this retrospective study, ACT between propofol-based TIVA by TCI and DES anesthesia were similar in patients undergoing open colorectal surgery. Importantly, this study found that prolonged extubation was associated with surgical times longer than 210 minutes, as well as age, in open colorectal surgery under TIVA or DES anesthesia.

Previous studies demonstrated that ACT, including exit from the OR after extubation, total OR time, and PACU time, may be affected by extubation time [7, 13, 14]. In other words, similar extubation time may contribute to equivalent PACU time, exit from OR after extubation, and total OR time in the same type of surgery. The results in our study, which showed no difference in extubation time and other ACTs between these two anesthetic techniques, are consistent with the above viewpoints.

Prolonged extubation is also an important factor that can decrease OR efficiency. Prolonged extubation time can slow work flow, with OR members idly waiting for extubation, and the surgeon having to wait longer for the next operation. Therefore, monitoring the incidence of prolonged extubation has been recommended as an economic measure [7]. Epstein and Dexter (2013) investigated the relationship between prolonged extubation and OR cost, and concluded that prolonged extubation time should be treated as resulting in proportionally increased variable costs [32]. In addition, 55.6% of the cases with prolonged extubation occurred during cases on regular workdays and in an OR with more than eight hours of cases and turnover [32,33]. Another study conducted by the same group demonstrated that the mean time from end of surgery to exit from the OR was at least 12.6 minutes longer in cases with prolonged extubation, and that the percentage of cases for which the extubation was prolonged (among anesthesia for intraperitoneal procedures in the lower abdomen) was 13.3% ± 0.5% [7]. In our present study, the percentage of prolonged extubation in the DES group was 14.2%, which is comparable to the above-mentioned study, while the percentage of prolonged extubation in the TIVA group was 9.7% (Table 3). There was no significant difference in the incidence of prolonged extubation between the TIVA and DES groups, which might be due to the groups’ similar BMI, gender, surgical times, and anesthesia times.

Studies have investigated the confounding risk factors for prolonged extubation in various surgical procedures. These factors included prone position, prolonged surgical time, significant blood loss, larger volume of crystalloid and colloid infusion, procedure, and surgeon [7,33]. Lai and Chan (2015) reported that DES anesthesia, longer anesthesia time, higher BMI, and shorter surgical time contribute to slower emergence in gynecologic laparoscopic surgery [15]. Chan and Lee (2015) demonstrated that the confounding factors that predicted awakening under TCI with propofol were age, sex, and length of surgery and anesthesia (total consumption dose of propofol and fentanyl) in a variety of surgeries [34].

In the present study, age, gender, BMI, and surgical time were analyzed to identify any association with prolonged extubation [15,34]. Our results illustrated that the odds ratio for prolonged extubation was 8.10 times and 64.7 times when surgical times were longer than 210 minutes and 240 minutes, respectively, compared with surgical times equal to or less than 210 minutes in open colorectal surgery. These findings implied that the best way to dramatically increase the efficiency of the operation room may be shortening the operation time through well-experienced operators, and applying new techniques or new operation protocols. Two studies further implied that longer-than-average anesthesia times strongly affect academic anesthesiology departments by increasing staffing costs and decreasing hourly productivity [35,36]. There is evidence that propofol may accumulate and washout slowly after continuous infusion in adults [37]. During lengthy surgical procedures, higher than necessary propofol infusion levels may accumulate and be redistributed from the fatty tissue and muscle to the plasma, which leads to delayed recovery. Inhaled DES is also redistributed in the fatty tissue and muscle, and may delay emergence in cases where the anesthesia time is increased [38]. Therefore, monitoring anesthetic depth to keep the hypnotic level within the recommended range improves anesthetic delivery and postoperative recovery from relatively deep anesthesia [39].

In the present study, although each age group was not at a significantly higher risk of prolonged extubation, compared to the youngest group, a modest linear trend was found in age after controlling for other covariates. Several physiological changes occur in elderly patients: the weight of the human brain decreases by about 10% with age, and the gray matter decreases more than the white matter [40], which may be more sensitive to anesthetics. Elderly patients have increased body fat with a greater volume of distribution, which might prolong the clinical effect of anesthetics. The decrease in renal and hepatic reserve in elderly patients may also prolong drug metabolism.

The anesthetics and techniques used should affect the extubation time. The amount of opioid and NDMRs in the DES group was significantly lower than in the TIVA group during surgical periods. This is reasonable, because volatile anesthetics may increase the potency of NDMRs [41] and demonstrate synergistic effects with opioids [42]. In addition, the reversal agents were not administered until spontaneous breathing had returned. Therefore, we believe the final neuromuscular blockade status and amount of reversal agents given were matched between groups.

Several studies have presented data that are consistent with our results on emergence time [20,29,30,43,44]. Dexter and Tinker (1995) demonstrated no significant difference in the time to following commands after discontinuation of DES versus propofol in orthopedic surgery, gynecological laparoscopic surgery, and ambulatory surgery [43]. In addition, four other studies showed that there was no significant difference in tracheal extubation time between a DES group and a propofol group in thyroid surgery under bispectral index (BIS) monitor [29], laparoscopic cholecystectomy (LC) [20], ontological surgery [44], and ear, nose and throat surgery [30]. However, these studies examined short-duration surgeries.

Wachtel and Dexter (2011) reported a meta-analysis comparing OR recovery times for DES anesthesia with propofol anesthesia, and the results showed that DES proportionally reduced the mean time to extubation by 21% relative to propofol [4]. Another recent meta-analysis of morbidly obese patients showed that patients given DES took less time (3.88 minutes) to be prepared for tracheal extubation than those given propofol [16]. These two meta-analyses, however, compared DES anesthesia with propofol under syringe pump instead of using a TCI machine; in addition, the surgical times were relative short. Both of these factors may have contributed to the different results reported. The TCI machine provides a function to estimate the effect-site concentration and the elimination time of propofol. In addition, TCI uses averaged pharmacokinetic models to control the infusion rate to regulate the calculated plasma concentration, rather than indirect control by adjusting the infusion rate [45], so the awakening time can be predicted [34]. Two studies have demonstrated that DES anesthesia shortened extubation time compared to TCI with propofol [8,46]. However, in these studies, nitrous oxide was used as an adjuvant to the anesthetics, which reduced the requirement of DES during the maintenance period and facilitated emergence.

Our previous studies showed that general anesthesia using the TCI system with propofol could achieve faster extubation than using DES anesthesia in various surgeries [10–15,38]. Different anesthetic manipulations before emergence in various types of surgical procedures might explain the differences in findings. For example, in breast [12] and gynecologic surgery [15], propofol was adjusted to a Ce of 2.0 ug/mL and the vapor of DES was changed to 5.0% at the beginning of wound closure. After gauze coverage, propofol and DES were discontinued, and the lungs were ventilated with 100% oxygen at a gas flow of 6 L/min. In ophthalmic surgery [13], DES or propofol was discontinued after surgery, and the lungs were ventilated with 100% oxygen at a fresh gas flow of 6 L/min. In spine surgery, we discontinued DES or propofol at the end of the operation or at the last three stitches of surgery. After turning the patients to a supine position, the lungs were ventilated with 100% oxygen at a fresh gas flow of 6 L/min [11,14]. In addition, we used closed-circuit anesthesia in the DES patients, which would prolong neuromuscular blockade and contribute to delay emergence [47].

There are several limitations to the present study. First, the proficiency of the anesthesiologist can affect the extubation time. In our study, six supervising anesthesiologists who have 5–15 years’ experience in clinical anesthesia conducted the anesthesia. Therefore, the variability might increase the risk of bias. Second, the final administration time of muscle relaxant, which is not mentioned in this study, could affect the extubation time. In our hospital, the final dose of muscle relaxant was administered before skin closure. Therefore, the efficacy was considered to be comparable. Third, regarding comparability and standardization of study groups, the retrospective design of this study may increase the risk of bias. Although the choice of anesthetic management was not randomly allocated, but was determined by the availability of the TCI devices, we found that there were no significant differences in various patient characteristics between the two groups. This study, performed under clinical conditions and with a large sample size, reflects more precisely the clinically-relevant benefit that may be expected with the use of new drugs, techniques, or devices. However, the results should be interpreted carefully. Patients in this study with lower blood pressure needed inotropes; experienced lengthy surgical times (> 8 hrs), blood loss > 2000 ml, and lower maintained anesthetic levels during the operation; tended to be ASA ≥ IV, had low BMI (< 16 kg/m^2^), or high BMI (female > 35 kg/m^2^ and male > 42 kg/m^2^); and were sent to the intensive care unit (ICU) without extubation postoperatively. Therefore, the above factors were not included in our calculations.

## Conclusions

In conclusion, our results demonstrate that the mean time to extubation in propofol-based TIVA by TCI is equivalent to desflurane anesthesia in open colorectal surgery. Prolonged surgical time and age contributed to prolonged extubation in this study.

## Supporting Information

S1 Table Characteristics and times from end of surgery to extubation reported in published studies comparing propofol to desflurane. To identify published manuscripts comparing extubation time after propofol and desflurane in humans, we searched PubMed on Sep 10, 2015 with the following terms in any field: (propofol OR Diprivan) AND desflurane AND (extubation OR extubate), limited to humans and our previous studies. N: sample size; TCI: target-controlled infusion; DES: desflurane; SD: standard deviation; LC: laparoscopic cholecystectomy; ENT: ear, nose and throat.
(DOCX)
