# Correction: Comparison of Anesthesia-Controlled Operating Room Time between Propofol-Based Total Intravenous Anesthesia and Desflurane Anesthesia in Open Colorectal Surgery: A Retrospective Study

**DOI:** 10.1371/journal.pone.0168310

**Published:** 2016-12-08

**Authors:** The PLOS ONE Staff

## Notice of Republication

This article was republished on November 3, 2016, to correct an error where an ORCID iD was erroneously assigned to Wei-Hung Chan. The publisher apologizes for this error.
