# Supplementary material for: Comparison of Anesthesia-Controlled Operating Room Time between Propofol-Based Total Intravenous Anesthesia and Desflurane Anesthesia in Open Colorectal Surgery: A Retrospective Study
Source: PLoS One. 2016 Oct 25;11(10):e0165407. doi: 10.1371/journal.pone.0165407 (PMC5079552; doi:10.1371/journal.pone.0165407)
Supplement: S1 Table — To identify published manuscripts comparing extubation time after propofol and desflurane in humans, we searched PubMed on Sep 10, 2015 with the following terms in any field: (propofol OR Diprivan) AND desflurane AND (extubation OR extubate), limited to humans and our previous studies. N: sample size; TCI: target-controlled infusion; DES: desflurane; SD: standard deviation; LC: laparoscopic cholecystectomy; ENT: ear, nose and throat. (DOCX) [file pone.0165407.s001.docx]

**Supporting Information**

**S1 Table. Characteristics and times from end of surgery to extubation reported in published studies comparing propofol to desflurane.**

| Authors (Reference) | n TIVA | n  DES | TCI | Titrated BIS or AEP | Propofol (min)  Mean (SD) Extubation | DES  (min)  Mean (SD) Extubation | Surgical  Procedure |
| --- | --- | --- | --- | --- | --- | --- | --- |
| Juvin(8) | 14 | 14 |  |  | 9.9 (6.5) | 6.9 (3.0) | General surgery (elderly) |
| Ashworth(17) | 30 | 30 |  |  | 5.6 (2.9) | 4.4 (1.5) | Ambulatory surgery |
| Song(18) | 40 | 40 |  |  | 8.9 (5.3) | 5.1 (3.3) | Ambulatory surgery |
| Juvin(19) | 11 | 12 |  | Yes | 13.2 (7.6) | 5.6 (1.4) | Laparoscopic gastroplasty (obesity) |
| Grundmman(20) | 25 | 25 |  |  | 5.5 (3.3) | 5.7 (2.5) | LC |
| Fredman(22) | 30 | 30 |  | Yes | 8.7 (3.8) | 6.1 (3.1) | Urologic surgery |
| Luginbuhl(23) | 40 | 40 |  |  | 10.5 (5.9) | 8.3 (6.1) | Gynecological surgery |
| Luginbuhl(23) | 40 | 40 |  | Yes | 6.8 (4.6) | 6.5 (4.1) | Gynecological surgery |
| Camci(25) | 25 | 25 |  | Yes | 6.9 (2.6) | 6.4 (2.6) | Ambulatory surgery |
| Erk(26) | 100 | 100 |  |  | 6.2 (3.2) | 2.3 (1.6) | LC |
| Gokce(27) | 20 | 20 |  |  | 6.8 (3.7) | 7.3 (3.4) | Septorhinoplasty |
| Horng(10) | 30 | 30 | Yes | Yes | 8.2 (3.0) | 13.7 (5.0) | Laparoscopic gynecological surgery |
| Chan(11) | 25 | 25 | Yes | Yes | 9.7(4.0) | 26.8(11.9) | Prolonged spine surgery |
| Chen(12) | 196 | 120 | Yes |  | 4.5(4.6) | 10.4(6.4) | Modified radical mastectomy |
| Wu(13) | 810 | 595 | Yes |  | 9.1(3.1) | 10.9(4.4) | Ophthalmic surgery |
| Lu(14) | 307 | 274 | Yes |  | 7.0(4.5) | 12.4(5.3) | Prolonged spine surgery |
| Lai(15) | 377 | 549 | Yes |  | 7.3(3.3) | 8.3(3.1) | Laparoscopic gynecological surgery |
| Pendeville(21) | 32 | 31 | Yes |  | 10.4 (3.0) | 10.2 (5.1) | Ambulatory oral surgery |
| Grottke(24) | 18 | 18 | Yes |  | 13.2 (2.3) | 7.5 (1.3) | Spinal surgery |
| Akkurt(28) | 30 | 30 | Yes |  | 6.4 (4.2) | 7.6 (0.7) | LC |
| Lee(29) | 38 | 38 | Yes | Yes | 6.3(2.9) | 5.8(1.7) | Thyroid surgery |
| Mahli(30) | 20 | 20 | Yes | Yes | 5.6(2.7) | 5.5(1.7) | ENT surgery |

To identify published manuscripts comparing extubation time after propofol and desflurane in humans, we searched PubMed on Sep 10, 2015 with the following terms in any field: (propofol OR Diprivan) AND desflurane AND (extubation OR extubate), limited to humans and our previous studies.

N: sample size; TCI: target-controlled infusion; DES: desflurane; SD: standard deviation; LC: laparoscopic cholecystectomy; ENT: ear, nose and throat
